# *Strongyloides stercoralis*: A Neglected but Fatal Parasite

**DOI:** 10.3390/tropicalmed7100310

**Published:** 2022-10-17

**Authors:** Viravarn Luvira, Tanaya Siripoon, Danabhand Phiboonbanakit, Kollawat Somsri, Dorn Watthanakulpanich, Paron Dekumyoy

**Affiliations:** 1 Department of Clinical Tropical Medicine, Faculty of Tropical Medicine, Mahidol University, Bangkok 10400, Thailand; 2 Division of Infectious Disease, Department of Medicine, Phramongkutklao Hospital, Phramongkutklao College of Medicine, Bangkok 10400, Thailand; 3 Vibhavadi Hospital, Bangkok 10900, Thailand; 4 Department of Helminthology, Faculty of Tropical Medicine, Mahidol University, Bangkok 10400, Thailand

**Keywords:** strongyloidiasis, *Strongyloides stercoralis*, immunocompromise, hyperinfection syndrome, disseminated strongyloidiasis, steroids

## Abstract

Strongyloidiasis is a disease caused by *Strongyloides stercoralis* and remains a neglected tropical infection despite significant public health concerns. Challenges in the management of strongyloidiasis arise from wide ranging clinical presentations, lack of practical high sensitivity diagnostic tests, and a fatal outcome in immunocompromised hosts. Migration, globalization, and increased administration of immunomodulators, particularly during the COVID-19 era, have amplified the global impact of strongyloidiasis. Here, we comprehensively review the diagnostic tests, clinical manifestations, and treatment of strongyloidiasis. The review additionally focuses on complicated strongyloidiasis in immunocompromised patients and critical screening strategies. Diagnosis of strongyloidiasis is challenging because of non-specific presentations and low parasite load. In contrast, treatment is simple: administration of single dosage ivermectin or moxidectin, a recent anthelmintic drug. Undiagnosed infections result in hyperinfection syndrome and disseminated disease when patients become immunocompromised. Thus, disease manifestation awareness among clinicians is crucial. Furthermore, active surveillance and advanced diagnostic tests are essential for fundamental management.

## 1. Introduction

Strongyloidiasis, a disease caused by *Strongyloides stercoralis*, continues to persist as a worldwide public health issue. However, the real burden of this disease is unknown, and studies have been performed in limited geographical areas and populations [1,2]. An estimated 370 million people with strongyloidiasis worldwide, with a prevalence between 10% and 40% of the population in tropical and subtropical countries, was dated back to 2013 [1,3]. Although the treatment of chronic strongyloidiasis is straightforward, infection diagnosis remains a challenge, resulting in prevalence being under-reported. A unique autoinfection stage during the *S. stercoralis* life cycle potentially causes lifelong parasitic infections [4], lingering analogous to a ticking time bomb, which eventually bursts into escalated and life-threatening hyperinfection or disseminated strongyloidiasis when patients experience immune response impairment [5].

## 2. Materials and Methods

A web-based search was performed via PubMed and Google Scholar. We included original articles, reviews, case reports, and short communications in English published from 1987 to 2021. The keywords included ‘*Strongyloides stercoralis*’, ‘strongyloidiasis’, ‘strongyloidiasis management’, ‘strongyloidiasis epidemiology’, ‘strongyloidiasis diagnosis’, ‘*Strongyloides stercoralis* serology’, ‘*Strongyloides* hyperinfection syndrome’, ‘disseminated strongyloidiasis’, and ‘strongyloidiasis and COVID-19′. Standard textbooks, guidelines, and article bibliographies were used for additional references.

## 3. Epidemiology

*Strongyloides* is a soil-transmitted nematode that is endemic, yet not confined to tropical and subtropical countries. The global prevalence of *S. stercoralis* has been estimated at 10–40% of the population in tropical and subtropical regions, equivalent to an estimated 30–100 million cases [6]. However, it is under-reported in areas in which low-sensitivity diagnostic tests are used. The epidemiology of *S. stercoralis* infection differs from that of other helminth infections because of its unique ability for reinfection in humans, also defined as autoinfection [7]. The estimated prevalence has varied among community-based, hospital-based, and refugee and immigrant studies [6]. Asudai et al. reported a 2019-meta-analysis of studies involving migrants worldwide and demonstrated a pooled strongyloidiasis seroprevalence of 12.2%: 17.3% from East Asia and the Pacific, 14.6% from sub-Saharan Africa, and 11.4% from Latin America and the Caribbean [8]. *S. stercoralis* accounts for most human infections, whereas *Strongyloides fuelleborni fuelleborni* or *S. fuelleborni kelleyi* account for rare infections in certain geographical regions, including Papua New Guinea, Thailand, and the Philippines [1,6,9]. Brazil and Thailand are major hotspots for strongyloidiasis, with a prevalence of 10.8–17% and 23.7–34.7%, respectively. Most studies in European countries have focused on refugees, immigrants, and travelers from endemic countries because *S. stercoralis* has a lower prevalence in developed countries and in urban areas of developing countries where fecal contamination in soil is scarce [1,6]. The estimated prevalence in developed countries is heterogeneous, depending on study type and population ranging from < 0.1% in high-income countries in temperate zones [10] to 12.4–14.8% among farm workers on the Mediterranean coast [11].

The Committee to Advise on Tropical Medicine and Travel (CATMAT) categorized the epidemiological risk for *Strongyloides* infection and exposure as follows [12]: high risk (>10%) for birth, residence, or long-term travel (defined as cumulative six-month exposure, or contact of skin with sand or soil in a high risk area during shorter-term travel) in Southeast Asia, Oceania, Sub-Saharan Africa, South America, and the Caribbean; moderate risk (3–10%) in Mediterranean countries, Middle East, North Africa, Indian sub-continent, and Asia; and low risk (<3%) in Australia, North America, or Western Europe [10,12,13]. The risk of developing complicated strongyloidiasis is more pronounced in patients with compromised cell-mediated immunity, especially in patients from endemic locations who later receive immuno-suppressive treatment [1].

## 4. Life Cycle

**Figure 1:**
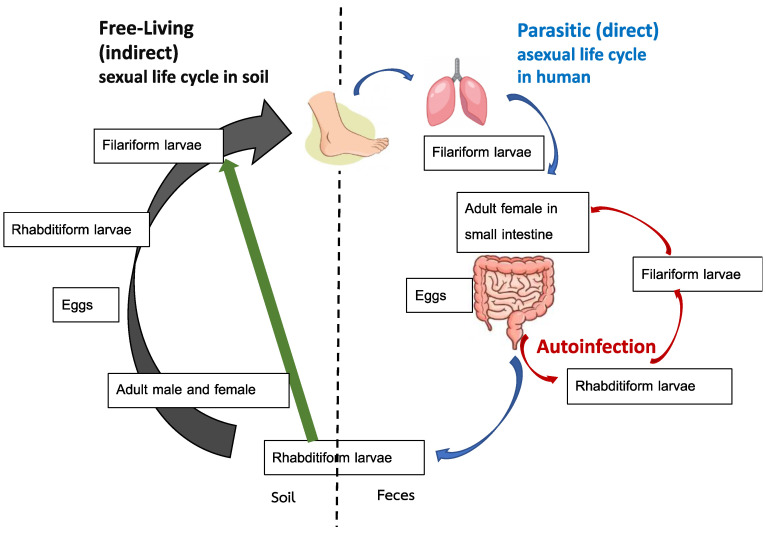
Life cycle of *Strongyloides stercoralis*. Adapted from reference no. [17].

**Figure 2:**
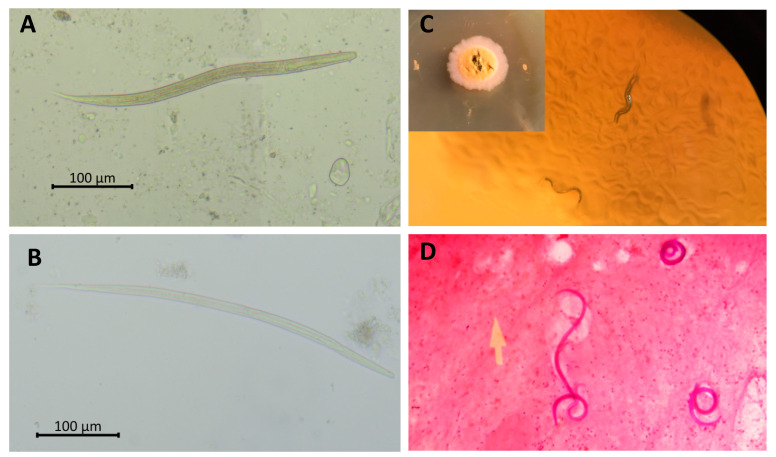
Parasitological detection of *Strongyloides stercoralis.* Rhabditiform (**A**) and filariform; (**B**) larvae of *S. stercoralis* fresh smear. Migrating rhabditiform larvae in agar plate culture; (**C**). Gram staining of filariform larvae in sputum of a patient with *S. stercoralis* hyperinfection syndrome (100×) (**D**) Picture; (**C**) courtesy of Poom Adisakwattana.

Comprehension of the unique life cycle associated with *S. stercoralis* is important for clinical evaluation and management. The life cycle can be divided into parasitic (direct) and free-living (indirect) stages, as shown in Figure 1. The indirect life cycle of *S. stercoralis* (Figure 1) initiates when rhabditiform larvae (Figure 2A) pass from stools to soil. Subsequently, the larvae can either directly transform into invasive filariform larvae, shown in Figure 2B, which is the infective stage, or develop and molt into the free-living phase and thrive as adults in soil. In the free-living stage, female and male adults exhibiting rhabditiform type characteristic esophagus mate and enable female adults to deposit eggs in soil. The eggs hatch into rhabditiform larvae that develop into filariform larvae, which is the infective stage.

*S. stercoralis* are soil-transmitted helminths. Humans are mainly infected via skin penetration of filariform larvae, especially through barefoot contact for the direct life cycle, or more rarely, through ingestion of contaminated food and drink. A serpiginous lesion (pruritus track) can be seen at the site of entry. Upon entry, larvae pass through the venous circulation and migrate to the heart and lungs. The filariform larvae, which later ascend the tracheobronchial tree, are expectorated into sputum and swallowed into the gastrointestinal (GI) tract, at which point molting and development into the adult stage are initiated. Subsequently, adults are embedded in the small intestinal mucosa, mainly in the duodenum. Female adults that asexually reproduce can be detected [4]. Finally, eggs hatch in the intestine and the rhabditiform larvae are excreted in stools.

The autoinfection life cycle occurs when the rhabditiform larvae develop into invasive filariform larvae prior to expulsion in stools. Subsequently, reinfection occurs during filariform larvae penetration into the intestinal mucosa (internal autoinfection) or perianal skin (external autoinfection) [14]. This unique autoinfection cycle enables *S. stercoralis* to cause persistent or even lifelong infection. The simultaneous detection of rhabditiform and filariform larvae in stools can reflect the autoinfection that usually occurs in hyperinfection syndrome or in immunosuppressed hosts [15]. Theoretically, the autoinfection life cycle spans 2–3 weeks. Thus, antiparasitic treatment should be administered in repeating 2–3-week intervals to ensure cure of strongyloidiasis [16].

## 5. Laboratory Diagnosis

At present, there is no gold standard diagnostic technique for *S. stercoralis* infection [18], and the available parasitological and serological diagnostic methods still possess limitations. The advantages and disadvantages of each diagnostic method are compared in Table 1. A combination of diagnostic techniques is recommended.

For parasitological detection, a single stool concentration examination has limited sensitivity to diagnose chronic strongyloidiasis because of low parasite load and irregular fecal shedding of larvae. Thus, repeated stool examination consisting of 3–7 specimens is recommended [17]. In contrast to chronic strongyloidiasis, an abundance of larvae can be obtained in stools through a simple smear from patients with hyperinfection or disseminated infection because of the high parasite load.

The Baermann funnel method is more sensitive than the single stool concentration technique. Although the Baermann funnel is less sensitive than the stool culture techniques for strongyloidiasis diagnosis, its quantification of parasite burden makes it suitable for utilization in study settings.

The culture techniques, such as Harada Mori culture, polyethylene tube culture, and agar plate culture have higher sensitivity than stool concentration techniques and are recommended as the laboratory investigations of choice. However, the disadvantages of the culture techniques are time consumption, high cost, availability limitations, and requirement for fresh stool samples [17,19]. For instance, agar plate culture, which is the most efficient stool culture technique, requires fresh stools and 2–3 days to detect migrating rhabditiform larvae on agar (Figure 2C). Stool culture techniques only enable detection of *Strongyloides* spp., *Trichostrongylus* spp., and hookworm larvae, thus requiring other stool examination techniques for the identification of concurrent parasite infections.

The presence of larvae in sputum and/or bronchoalveolar lavage indicates hyperinfection syndrome, and simple fresh smear and special staining can be performed (Figure 2D). A high index of suspicion is required, and bedside sputum examination is essential for the diagnosis of strongyloidiasis hyperinfection syndrome. Tissue biopsy is required to diagnose ectopic foci (organs not involved in the life cycle) of *S. stercoralis* in disseminated infection. Furthermore, rhabditiform larvae can be found in duodenal biopsy when patients (especially transplant hosts) undergo upper GI endoscopy for other reasons [20].

Eosinophilia is common in strongyloidiasis, but it is usually mild (5–15%) and nonspecific [17]. A study in an Aboriginal community in the endemic area of North Australia reported that eosinophilia had 60.9% sensitivity, 71.1% specificity, 54.6% positive predictive value, and 76.1% negative predictive value for *S. stercoralis* infection [21]. The presence of eosinophilia may suggest parasitic infections including *S. stercoralis* but the absence of eosinophilia cannot rule out strongyloidiasis. Moreover, eosinophilia tended to present less in *S. stercoralis* infection in immunocompromised individuals [22].

In terms of serological methods, techniques used to detect antibody response to *S. stercoralis* include enzyme-linked immunosorbent assay (ELISA), indirect agglutination, indirect immunofluorescence, and western blotting (immunoblotting). Their respective sensitivities and specificities depend on the types of antigens used and the detected immunoglobulins. In principle, serology is more sensitive than fecal-based methods. Serology is effective for diagnosis of stronyloidiasis in people residing in endemic areas, and immigrants who require a screening test prior to immunosuppressive treatment, and for use in seroepidemiological studies. Furthermore, serology is useful in post-treatment follow-up and in monitoring outcomes of public health control intervention programs [17,23,24,25]. The drawbacks of serology include low sensitivity in patients with impaired immunity [26,27] and cross-reactivity with other helminthiases, such as filariasis, ascariasis, and schistosomiasis [17,19]. However, cross-reactivity is dependent upon prepared antigen type and additional diseases present in respective laboratory studies. Our center, the Faculty of Tropical Medicine, Mahidol University is a center of excellence in parasite diagnostic serology. Our indirect IgG-ELISA diagnosis of strongyloidiasis, prepared from a modified molecular weight cut-off antigen, yielded a sensitivity and specificity of 96% and 94%, respectively, in immunocompetent hosts, and 42.9% and 96.3%, in immunocompromised hosts [27,28]. Recently, several sensitive and specific point-of-care serological tests for strongyloidiasis were developed and showed sensitivities and specificities of 91.3–93.3% and 83.8–100%, respectively [29,30,31]. The rapid point-of-care test also worked well in a field study, with 82% sensitivity and 96% specificity [32].

For a decade, the molecular techniques of polymerase chain reaction (PCR) and loop-mediated isothermal amplification (LAMP) have been developed for diagnosis of strongyloidiasis with varying results [33,34]. Apart from stool examination, the molecular techniques retain compatibility for other clinical specimens including sputum, blood, urine, bronchoalveolar lavage, and intestinal aspiration [33,35]. A meta-analysis reported that high specificity with limited sensitivity validates PCR as more suitable for diagnostic confirmation, as opposed to an initial screening test for strongyloidiasis [36]. Although LAMP offers a rapid and economic testing alternative, it appears less effective for *S. stercoralis* detection in clinical specimens when compared with PCR [33].

## 6. Clinical Syndromes

*S. stercoralis* can cause a wide spectrum of disease presentations depending on the host’s immunity and parasite load. The clinical syndromes include acute strongyloidiasis, chronic strongyloidiasis, hyperinfection syndrome, and disseminated infection. Strongylodiasis can be simply classified as “uncomplicated” (acute and chronic strongyloidiasis) or “severe or complicated” (hyperinfection syndrome and disseminated infection) [17].

### 6.1. Acute Strongyloidiasis

This syndrome is rarely diagnosed. It is mainly reported in travelers who returned from endemic areas [5,37]. The symptoms at this stage are from reactions at the site of larval entry and migration, which normally occur immediately to several weeks post-infection. Skin manifestations include a serpiginous lesion (urticarial track with severe pruritus) at the entry site; the lung migration of parasites can result in dry cough and wheezing or even the classic Loeffler-like syndrome [38]. Lastly, GI symptoms begin when the parasites reach the intestine ~2 weeks after infection [4]. At this stage, serological testing is usually negative, and diagnosis is based on rhabditiform larvae detection in stools which is normally found 2–4 weeks after infection [39].

### 6.2. Chronic Strongyloidiasis

**Figure 3:**
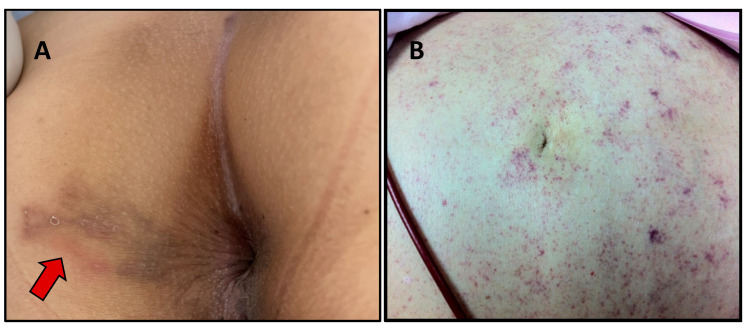
Skin lesions that can be found in strongyloidiasis. Larva currens in the perianal area (arrow) (**A**) and periumbilical parasitic thumbprint purpura in a patient with disseminated strongyloidiasis (**B**). Picture (**A**) courtesy of Than Narkwiboonwong.

Most people with chronic strongyloidiasis are asymptomatic or have mild symptoms. The common GI symptoms include abdominal bloating, epigastric pain that worsens by eating, intermittent vomiting, diarrhea, constipation, and borborygmus. Larva currens, a rapid intradermal migration of larvae (5–15 cm/h), is the pathognomonic skin lesion (Figure 3A) [38]. Other skin manifestations include urticarial and recurrent maculopapular rashes. A meta-analysis reported that the significant symptoms associated with chronic strongyloidiasis were abdominal pain, diarrhea, and urticaria [2].

### 6.3. Hyperinfection Syndrome

Hyperinfection syndrome is defined by an increase in parasite load from the autoinfection life cycle, usually (but not always) caused by impairment of immune status. Thus, the larvae in non-disseminated hyperinfection syndrome are confined to organs directly involved in the *S. stercoralis* life cycle, which includes the GI tract, peritoneum, and lungs. Skin lesions in hyperinfection syndrome consist of larva currens, petechial, and purpuric rashes that are commonly found in the lower trunk, thighs, and buttocks [4].

A retrospective study reported the common clinical manifestations of *Strongyloides* hyperinfection syndrome which included fever (80.8%), respiratory (88.6%), and GI (71.2%) symptoms [40]. The pulmonary symptoms of hyperinfection syndrome included cough, shortness of breath and asthma-like presentations; acute respiratory distress can also occur [41]. However, diagnostic difficulties may arise as immunocompromised patients often possess underlying pulmonary diseases, or the symptoms might be masked by secondary infections. Chest radiography is often variable. The classic bilateral or focal interstitial infiltrates can be detected. However, the consolidations and abscesses can occur especially with concurrent bacterial pneumonia. Sputum examination demonstrates filariform or rhabditiform larvae and even occasionally eggs [4].

### 6.4. Disseminated Infection

Disseminated infection describes the migration of larvae to organs beyond the range of the pulmonary autoinfective cycle. The larvae are found in ectopic sites including the skin, liver, brain, heart, and urinary tract. Periumbilical parasitic thumbprint purpura is a rare skin presentation classically found in disseminated strongyloidiasis (Figure 3B) [42]. This particular skin lesion results from the penetration of larva into the skin.

Concurrent bacterial and fungal infections, mostly from enteric pathogens, often occur in hyperinfection and disseminated infection, and the clinical presentations include bacteremia, peritonitis, and pneumonia [17]. Blood and cerebrospinal fluid cultures were positive for bacteria in 29.1% and 15.2%, respectively, of 151 patients with severe strongyloidiasis [43]. Shock and respiratory failure were reported in up to 57.3% and 67.9%, respectively, of patients with hyperinfection syndrome [41].

## 7. Strongyloidiasis in Immunocompromised Patients

The immunocompromised conditions associated with severe strongyloidiasis are mainly defects in the cell mediated immune response. The most common condition is corticosteroid treatment. One study reported that the mean corticosteroid dose in patients with severe strongyloidiasis was 52 ± 42 mg prednisolone equivalent per day, and the duration ranged from four days to 20 years [43]. Other treatments associated with severe strongyloidiasis are chemotherapy, cyclosporine, azathioprine, total body irradiation, and transplantation. Diseases with a high risk of severe strongyloidiasis are hematological malignancy, human T-cell lymphotropic virus type 1 (HTLV-1) infection, malnutrition and hypogammaglobulinemia [4,26]. The risk of severe strongyloidiasis in people living with HIV has been debated; disseminated strongyloidiasis was removed from the list of opportunistic infections because corticosteroid use in severe *Pneumocystis* pneumonia was suspected to be a confounding factor [1,4]. However, the prevalence of strongyloidiasis is high in patients with HIV infection, especially among residents and travelers from endemic areas. A previous study reported up to 25% seroprevalence among antiretroviral-naive HIV patients with CD4 count ≤ 100 cells/µL [44].

The real impact of strongyloidiasis among immunocompromised individuals is unknown and it is believed to be under-reported because of inadequate screening and difficulties in diagnosis. The reported prevalence varies from 3% to 23.0% depending on the population and method of testing [27,45,46,47]. The clinical presentation of strongyloidiasis varies from asymptomatic in chronic strongyloidiasis to a fatal disseminated syndrome, depending on host immunity. A cross-sectional study in immunocompromised patients in Thailand revealed a strongyloidiasis prevalence of 6.7%; of which 62.5% of the cases were asymptomatic or chronic strongyloidiasis [27].

Transplantation patients can develop a severe form of strongyloidiasis from their previous infections or new infections during transplantation. Donor derived *S. stercoralis* infection was documented in solid organ transplant (SOT), especially in renal transplantation [20,48,49], leading to recommendations for *S. stercoralis* screening in both donors and recipients. Complicated strongyloidiasis developed earlier and had an increased fatality rate in hematopoietic stem cell transplantation (HSCT) when compared with SOT as a result of supplementary intensive immunosuppression [20]. In SOT, complicated strongyloidiasis usually occurred within three months after transplantation [20]. Cyclosporin metabolite produces anti-parasitic effects against *Strongyloides* spp. Thus, reduced use of cyclosporin-based regimens may result in increased prevalence of severe complicated strongyloidiasis [50,51].

The critical challenge in the diagnosis of severe strongyloidiasis in immunocompromised patients is the potential absence of symptoms and presentations (caused by poor immune response) until the patients reach the full-blown stage. A seroprevalence study among renal transplant recipients reported a significant decrease in IgG titer in repeatedly transplanted patients, suggesting decreased antibody response in more immunosuppressed hosts [47]. Thus, serological testing for strongyloidiasis appears to decrease in sensitivity for immunocompromised patients [26]. Our previous comparative diagnostic study of strongyloidiasis among hospitalized immunocompromised patients in Bangkok, Thailand, reported a decrease in sensitivity of IgG obtained by indirect-ELISA testing from 96% to 42.9% while specificity remained high [27]. The stool agar plate culture remains the investigation method of choice for this group of patients [27].

**Figure 4:**
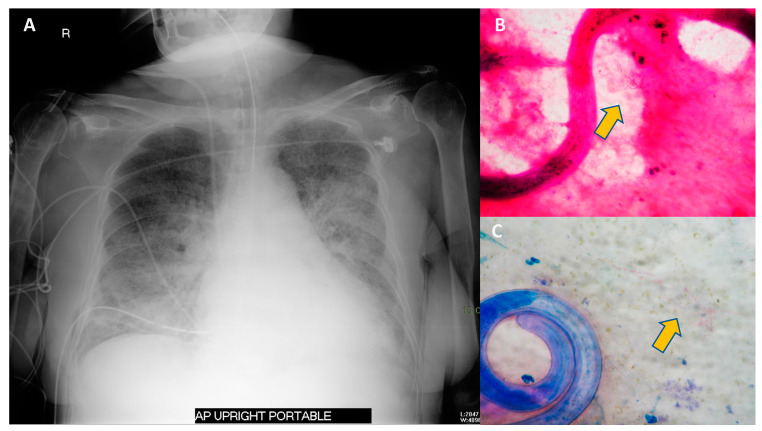
Chest radiography and sputum examination from a case of *S. stercoralis* hyperinfection syndrome in an immunocompromised patient. A 75-year-old Thai woman with temporal arteritis had been treated with prednisolone 20 mg/day for 4 months. She developed fever and diarrhea for 2 days prior to respiratory failure. The chest radiography revealed bilateral patchy infiltration (**A**). Sputum examination with Gram staining (**B**) and Modified Acid-Fast staining (**C**) revealed filariform larvae of *S. stercoralis* and a positive branching filamentous organism (arrow), indicating *Nocardia* species. Blood cultures grew *Escherichia coli*. She was diagnosed with *S. stercoralis* hyperinfection syndrome with concurrent Gram-negative bacteremia and pulmonary nocardiasis. She was treated with broad-spectrum cephalosporin, cotrimoxazole and ivermectin.

Immunocompromised hosts with secondary bacterial and fungal infections have poor outcomes in complicated strongyloidiasis. A mortality rate of 69% was reported among immunocompromised patients with severe strongyloidiasis in the USA [52]. An example of *S. stercoralis* hyperinfection syndrome in an immunocompromised patient is shown in Figure 4.

## 8. Management

### 8.1. Management of Uncomplicated Strongyloidiasis

The antiparasitic drugs effective against strongyloidiasis include ivermectin, benzimidazole compounds (thiabendazole, albendazole and mebendazole), and pyrvinium pamoate. However, most anti-parasitic agents cannot kill migrating larvae and eggs in autoinfection. Thus, repeating the regimen at 2–3-week intervals is usually recommended to eradicate autoinfection. The clinical studies of treatment for chronic uncomplicated strongyloidiasis are summarized in Table 2.

Albendazole shows less efficacy than thiabendazole in the treatment of chronic strongyloidiasis, although it is better tolerated. Albendazole also has activity against a broad range of intestinal helminths.

Nowadays, ivermectin is the drug of choice for uncomplicated strongyloidiasis. A recent phase III randomized control trial revealed that a single dose of ivermectin (200 µg/kg) was sufficient for treatment of uncomplicated strongyloidiasis [65]. In addition to regimen simplicity, this drug is also generally well tolerated in patients. The common adverse events of ivermectin are abnormal liver enzymes (13.6%), itching (1.8–11.8%), headache (8.8–10.6%), loose stools (1.2–9.8%), cough (6.7%), fever (6.3%), and fatigue (0.8–5.9%) [16].

Moxidectin is a veterinary antiparasitic drug that has FDA approval for the treatment of human onchocerciasis and is in line for clinical trials in strongyloidiasis. A single dose of 8 mg veterinary form moxidectin was safe and demonstrated non-inferior efficacy to ivermectin for treatment of uncomplicated strongyloidiasis in a phase II randomized controlled trial [64]. A randomized controlled trial with ascending doses supported a single 8-mg dose of human moxidectin tablets (similar dose for onchocerciasis) for treatment of chronic strongyloidiasis [66]. The advantages of moxidectin over ivermectin include more convenience as a single oral dose independent of patient weight, less neurotoxicity, and a larger volume of distribution with a longer half-life, which might be beneficial towards eradication of auto- and re-infection [66,67]. The drug was also proposed as an alternative in cases with ivermectin failure [67].

### 8.2. Management of Complicated Strongyloidiasis

The treatment of complicated strongyloidiasis is based on case reports and series. The systematic analysis of case reports showed better outcomes for ivermectin treatment when compared with other single regimen treatments [68]. Although there are no standard guidelines for treatment of severe strongyloidiasis, ivermectin at 200 µg/kg/day until resolution of clinical syndromes and absence of parasite detection in three consecutive specimens has been recommended [4,16,20]. Additionally, continuing treatment until negative fecal culture of *S. stercoralis* for two weeks has been suggested to eradicate autoinfection [4,69].

Due to the serious condition of cases of complicated strongylodiasis occurring in nature, multiple antiparasitic drugs or multiple routes of ivermectin have been applied in combination with oral ivermectin [70]. Subcutaneous injection of a veterinary parenteral form of ivermectin has been used as a salvage regimen or in patients with absorption problems [71]. The common dosage of parenteral ivermectin was 200 µg/kg/day (range: 75–200 µg/kg/day) and the duration of treatment was in the range of 3–22 doses [16,71]. Parenteral ivermectin is not authorized for human usage and severe neurotoxicity has been reported in patients with complicated strongyloidiasis [72,73]. Thus, patients’ consent before prescription is suggested. To date, there is no report of moxidectin treatment for complicated strongyloidiasis in immunocompromised individuals.

Apart from antiparasitic treatment, the key management of complicated strongyloidiasis is restoring host immunity. The use of immunosuppressive agents needs to be minimized. Furthermore, the concurrent bacterial and fungal infection must be evaluated and empirically treated.

### 8.3. Follow-Up after Treatment

Both clinical and laboratory evaluation should be performed after treatment. Laboratory evaluations include stool examination, complete blood count and serology. In severe forms of strongyloidiasis, stool follow-up examination should be performed for at least two weeks to ensure eradication [69]. Eosinophilia usually declines to normal levels within one month while serology requires 6–12 months. Therefore, sequential testing every 3–6 months for two years is recommended [16,23]. Screening for HTLV-1 infection is advised in cases of treatment failure [38].

## 9. Screening and Prevention

To prevent severe forms of strongyloidiasis in immunocompromised patients, vigilant screening before and during immunosuppressive treatment is essential. A combination of tests, including stool concentration examination, stool culture, and serological methods, is recommended [4,27,74]. Some experts have suggested that combination screening should include stool PCR [75]. In cases where sensitive screening tests are not available, pre-emptive treatment with ivermectin is suggested [68,76]. Parasites can be transmitted via SOT, and there is a high prevalence of strongyloidiasis among donors from endemic areas [77]. Therefore, screening tests are recommended in both recipients and donors before transplantation [51,78]. The infected living donors should be cured prior to transplantation while recipients of untreated infected donors require empiric therapy after transplantation and close monitoring [51].

In addition to screening, routine empiric treatment of strongyloidiasis with ivermectin in immunocompromised patients or prior to immunosuppressive treatment has been the recommendation, especially in endemic areas, although no standard regimen or significant evidence-based study has been reported [79,80]. Re-infection is common in endemic areas. Thus, routine periodic deworming has been suggested [78].

In immunocompetent individuals, screening for strongyloidiasis with a preference for serological techniques is recommended in immigrants or long-term travelers (>1 year) from an endemic area [76].

Contact isolation should be applied in all strongyloidiasis cases in addition to screening of all patients’ family members. Patient education that focuses on personal hygiene (using latrines and wearing shoes in endemic areas) should be provided to people at risk (immunocompromised individuals) and the general population.

In endemic areas, community control was successful through proactive case screening and pharmacological treatment regardless of environmental sanitation improvements [81,82]. Mass administration of ivermectin for strongyloidiasis or other parasitic infections has produced beneficial effects toward sustained reduction in prevalence [83,84].

## 10. Strongyloidiasis and COVID-19

Dexamethasone has been shown to reduce mortality in hospitalized patients with moderate-to-severe COVID-19 [85]. Although most immunocompetent patients have chronic asymptomatic strongyloidiasis, immunocompromised patients, especially those undergoing corticosteroid therapy, can progress to advanced disease, such as disseminated strongyloidiasis and hyperinfection syndrome [86,87]. The prevalence of COVID-19 and strongyloidiasis coinfection is unclear. Pereira and colleagues summarized the reported cases, in which the majority were male, with an average age of 61.3 years, were discharged from hospital, and then returned with skin presentations and symptoms followed by respiratory and GI symptoms [86,88,89,90,91,92]. Symptoms of severe COVID-19 are mainly cough (68.9%), fever (71.6%), dyspnea (71.2%), and diarrhea (20%), while those of *Strongyloides* hyperinfection syndrome are cough, fever (80.8%), dyspnea or wheeze (88.6%), GI symptoms (71.2%), and disseminated larva currens [93]. Chronic strongyloidiasis and COVID-19 could become commonplace, especially in endemic low- and middle-income countries. Thus, clinicians should evaluate underlying chronic strongyloidiasis infection independent of signs and symptoms, epidemiology, and other behavioral risk factors prior to corticosteroid initiation [87]. According to the 2016 CATMAT recommendation of risk stratification, a test-and-treat strategy is suggested for mild COVID-19 where serological testing is available. Presumptive ivermectin treatment is considered reasonable for moderate- to high-risk patients who are candidates for corticosteroids (equivalent to 20 mg/day prednisolone for ≥ 2 weeks) and have not previously received testing or treatment [12,86,87]. Although *Strongyloides* serology and stool tests are ideally recommended prior to initiation of immunosuppression, if immediate circumstances limit feasibility, the tests should subsequently be performed as soon as possible. Regarding a limited supply of ivermectin, presumptive ivermectin treatment for COVID-19 patients should be reserved for: (i) empiric therapy for patients with very high epidemiological risk; (ii) patients with asymptomatic strongyloidiasis with positive serology; (iii) *Strongyloides* hyperinfection or disseminated disease; and (iv) patients with symptomatic *Strongyloides* infection or high risk of progression to *Strongyloides* hyperinfection or disseminated disease [13]. Potential, but not high-risk, patients should be monitored for clinical deterioration upon immunosuppression. Prompt investigation with stool microscopy, respiratory samples examination, or culture should be undertaken if *Strongyloides* hyperinfection is suspected [93].

## 11. Conclusions

The unique nature of *S. stercoralis* results in clinical practice challenges, including diagnostic complexities, a broad spectrum of disease presentations depending on the host’s immunity, and corresponding difficulties caused by treatment complications. The diagnosis of acute and chronic strongyloidiasis is difficult because of the non-specific presentations and low parasite load. All available investigations have limited sensitivity. Thus, the combination of parasitological and serological methods is recommended. The gold standard regimen is a single dose of ivermectin. Moxidectin has the potential to become the drug of choice in the future. In contrast, poor host immunity, high parasite burden from autoinfection, and concurrent bacterial and fungal infection lead to treatment complexity and high mortality in hyperinfection and disseminated strongyloidiasis. Increases in traveling and migration, as well as advances in immunosuppressive treatment, particularly during the COVID-19 pandemic, have raised the impact and awareness of strongyloidiasis. Active surveillance and further research regarding diagnostic techniques are required to reveal the real burden of this under-reported disease. The standard recommendations need to be strengthened for screening and prophylactic strategies in immunocompromised patients, individuals undergoing immunosuppressive treatment, as well as immigrants and long-term travelers from endemic areas.

## Figures and Tables

**Table 1:** Summary of diagnostic tests for strongyloidiasis.

Method	Advantages	Disadvantages
**Stool concentration**	-Practical in clinical setting -Able to detect other parasites	-Variation in sensitivity (operator dependent)
**Baermann funnel**	-Quantify parasite burden and suitability for low parasite load	-Requires large amount of stool
**Stool culture**	-High sensitivity	-Cannot detect other parasites -Requires fresh stool, time consuming -Limited availability
**Serology**	-More sensitive than fecal-based methods, suitable for screening and diagnosis based on well-prepared antigens -Can be used in both endemic area residents and immigrants -Screening prior to immunosuppressive treatment -Seroepidemiological studies -Use in follow-up studies (decrease 6–12 months after treatment)	-Low sensitivity in patients with impaired immunity and low immune response -Limited specificity for cross-reactivity with other helminth infections (such as filariasis, ascariasis and schistosomiasis) based on antigen used and detected immunoglobulin
**Molecular**	-Applicable to other clinical specimens -High specificity (with limited sensitivity), suitable for confirmation tests	-Limited availability -Variation in sensitivity and specificity depending on techniques used

**Table 2:** Clinical studies in treatments of chronic uncomplicated strongyloidiasis.

Study	Population	Study Design	Follow-Up Period	Regimens	Cure/Total N (%)		Remarks
Pungpak S, et al., 1987 [53]	Thai adults without co-morbidity	Controlled trial, open label	15, 30 days	Albendazole 400 mg/day for 3 days	8/11 (72.2)		
				Albendazole 400 mg/day for 3 days with repeated regimen 1 week later	19/19 (100)		
Archibald LK, et al., 1993 [54]	British ex-Far East prisoners of World War II	Prospective cohort	6–9 months, using stool tests and/or serology	Albendazole 400 mg twice daily for 3 days	35/47 (75)		-
Pitisuttithum P, et al., 1995 [55]	Thai adults without co-morbidity	RCT, open label	3 weeks	Albendazole 400 mg twice daily for 5 days	18/23 (94.7)		*p* = 0.183
				Thiabendazole 1 g twice daily for 5 days	12/12 (100.0)		
Shikiya K, et al., 1994 [56]	Japanese patients	Prospective study	No data	Ivermectin 6 mg in 2 doses, 2 weeks interval	108/125 (86.4)		-
Datry A, et al., 1994 [57]	French adults without co-morbidity	RCT, open label	3 months	Ivermectin 150–200 µg/kg, single dose	24/29 (82.8)		*p* < 0.01
				Albendazole 400 mg/day for 3 days	9/24 (37.5)		
Gann PH, et al., 1994 [58]	Southeast Asian refugees in the United States	RCT, open label	≥3 months	Ivermectin 200 µg/kg, single dose	16/16 (100)		-
				Ivermectin 200 µg/kg/day for 2 consecutive days	17/18 (94.4)		
				Thiabendazole 50 mg/kg/day for 3 days	18/19 (94.7)		
Toma H, et al., 2000 [59]	Japanese adults, positive rate of HTLV-1 infection was 29.4%	Controlled trial, open label	1 year	Pyrvinium pamoate 5 mg/kg/day for 3 days	14/60 (23.3)		-*p* < 0.001; pyrvinium pamoate vs. albendazole -*p* < 0.001; albendazole vs. ivermectin -Significantly decreased cure rate in HTLV-1 infection subjects
				Albendazole, 400 mg/day for 3 days	65/84 (77.4)		
				Ivermectin, 6 mg in a single dose	65/67 (97.0)		
Igual-Adell R, et al., 2004 [60]	Spanish adults	Retrospect-ive study	3 months	Ivermectin 200 µg/kg, single dose	17/22 (77.3)		-
				Ivermectin 200 µg/kg/day for 2 consecutive days	35/35 (100)		
				Thiabendazole 50 mg/kg/day for 5 days	25/31 (78)		
Suputtamongkol Y, et al., 2008 [61]	Thai adult patients with concomitant medical illness	RCT, open label	4 weeks	Veterinary (parenteral formulation) ivermectin 200 µg/kg, orally, single dose	16/21 (76.2)		*p* = 0.029
				Albendazole 800 mg/day for 7 days	8/21 (38.1)		
Bisoffi Z, et al., 2011 [62]	Italian and immigrant adults without co-morbidity	RCT, open label, phase III	3–6 months, Using both stool tests and serology	Ivermectin 200 µg/kg, single dose	60/106 (56.6)		*p* = 0.53
				Thiabendazole 50 mg/kg/day for 2 days	48/92 (52.2)		
Suputtamongkol Y, et al., 2011 [63]	Thai adult patients with concomitant medical illness	RCT, open label	1 year	Ivermectin 200 µg/kg, single dose	30/31 (96.8)		*p* = 0.006; albendazole vs. ivermectin
				Ivermectin 200 µg/kg/day, two doses, given 2 weeks apart	27/29 (93.1)		
				Albendazole 800 mg/day for 7 days	19/30 (63.3)		
Barda B, et al., 2017 [64]	Loa healthy people aged > 12 years	Non-inferior, RCT, single-blind, phase II	21 days	Moxidectin 8 mg, single dose	59/63 (93.7)		
				Ivermectin 200 µg/kg, single dose	59/62 (95.2)		
Buonfrate D, et al., 2019 [65]	European aged > 5 years and weight > 15 kg without immunosuppr-ession	Multileft, superior RCT, open label, phase III	1 year using stool tests and/or serology	Ivermectin 200 µg/kg/day, 4 doses (day 1, 2, 15, 16)	102/118 (86)		*p* = 0.75 Early terminated trial from futility
				Ivermectin 200 µg/kg, single dose	96/113 (85)		
Hofmann D, et al., 2021 [66]	Loa adult (aged 18–65 years) community members	Single-blinded, RCT, parallel-group, placebo-controlled, dose-ranging, phase 2a trial	28 days after treatment		Cure/total N (%)	Predicted cure rate% (95% CI)	
				Moxidectin 2 mg, single dose	22/30 (73)	75% (59–87)	-Stratify participants based on baseline -*S. stercoralis* infection intensities: light, moderate, heavy
				Moxidectin 4 mg, single dose	26/29 (90)	83% (76–88)	
				Moxidectin 6 mg, single dose	27/32 (84)	86% (79–90)	
				Moxidectin 8 mg, single dose	24/29 (83)	87% (80–92)	
				Moxidectin 10 mg, single dose	29/30 (97)	88% (80–93)	
				Moxidectin 12 mg, single dose	26/30 (87)	88% (80–93)	
				Placebo	4/29 (14)	14% (5–31)	

## Data Availability

Not applicable.
